# Study on the Differences in the Thirst-Quenching Effects of Different Beverages Supplemented Before Exercise: A Randomized Crossover Trial

**DOI:** 10.3390/nu17050760

**Published:** 2025-02-21

**Authors:** Jieying Gao, Yuchen Wang, Xiao Ren, Ying Nie, Yanmi Li, Yimin Zhang, Shuxian Huang, Dazhou Zhu

**Affiliations:** Institute of Food and Nutrition Development, Ministry of Agriculture and Rural Affairs, Beijing 100081, China; 82101225564@caas.cn (J.G.); 82101232184@caas.cn (Y.W.); 82101231145@caas.cn (X.R.); nieying01@caas.cn (Y.N.); liyanmi@stu.cdu.edu.cn (Y.L.); zhangyimin@stu.cdu.edu.cn (Y.Z.); 821012450674@caas.cn (S.H.)

**Keywords:** beverages, hydration, fluid intake

## Abstract

**Background/Objectives:** Different beverages may vary in their effectiveness at quenching thirst. This study aims to explore the impact of pre-exercise consumption of different types of beverages on thirst relief, providing scientific evidence to guide the selection of the most suitable beverage type. **Methods:** A randomized crossover design was used, recruiting 13 healthy male college students as participants. Each participant completed five exercise trials, with a 7-day interval between trials. In random order, participants consumed 6 mL/kg body weight of water, carbonated beverage, juice, electrolyte drink, or tea before exercise in each trial. Blood, saliva, and urine samples were collected before and after exercise; body weight was measured, and thirst sensation was recorded. **Results:** Body weight significantly decreased in all groups post-exercise (*p* < 0.05), with no significant differences between the beverage groups (*p* > 0.05). Post-exercise, serum Na^+^ concentrations significantly decreased in all beverage groups, with the electrolyte drink group showing a significantly different change compared to the other groups (*p* < 0.05). Serum K^+^ concentrations significantly increased post-exercise only in the electrolyte drink group (*p* < 0.05). No consistent trend was observed in the changes in serum Ca^2+^ concentrations before and after exercise. Serum Cl^−^ concentrations post-exercise were significantly lower than pre-exercise in all groups except the electrolyte drink group (Group E) (*p* < 0.05). All five hydration protocols resulted in a decrease in plasma volume. There was no consistent pattern in the changes in urine osmolality before and after exercise. Salivary osmolality significantly increased post-exercise in all groups (*p* < 0.05). In terms of subjective thirst, the water supplementation group had the highest score. **Conclusions:** This study indicates that electrolyte drinks are more effective in maintaining physiological balance, while water is most effective in alleviating subjective thirst. The impact of different beverages on subjective thirst did not fully align with changes in physiological markers, suggesting that future research should comprehensively evaluate the relationship between subjective sensations and physiological changes.

## 1. Introduction

Thirst is primarily induced by factors such as physiological conditions or subjective behaviors, which, in turn, trigger the desire to rehydrate [1]. Thirst is primarily regulated by the hypothalamus in the brain and the kidneys. When fluid intake is insufficient, or dehydration becomes excessive, it excites the neural nuclei of the thirst center, triggering the sensation of thirst and stimulating subjective fluid intake behavior. When dehydration reaches 1% to 2% of body weight, the thirst drive prompts drinking. However, there is considerable variability between individuals in their perception of thirst and fluid needs [2]. During the relief of thirst, different beverages exhibit varying effects due to differences in their composition. Therefore, how to integrate subjective sensations with objective physiological markers to select an effective beverage for quenching thirst has become a key focus of related research. Silvino, V. O. et al. [3] investigated the effects of electrolyte beverages on the physical performance and hydration status of recreational runners by examining physiological indicators such as resting heart rate, urine volume, urine specific gravity, and serum electrolyte concentrations before and after exercise. Danniela García-Berger et al. [4] explored the effects of consuming skimmed milk and isotonic beverages before exercise on the fluid balance of cyclists by examining changes in indicators such as body weight and urine color before and after a road cycling time trial. On the other hand, clinical studies evaluate patients’ subjective thirst sensations through scales. Yang Man et al. [5] used thirst numerical rating scales, the Hospital Anxiety and Depression Scale, and other assessment tools to conduct face-to-face surveys on patients’ thirst status before digestive endoscopy procedures. Wei Chen et al. [6] employed the Numerical Rating Scale (NRS) to assess patients’ thirst levels from the pre-operation period from the 1st to the 5th day post-operation. Mei Bengang et al. [1] used the NRS to evaluate the degree of thirst in two groups of patients before and after interventions. Although these studies provide some references, existing research primarily focuses on the medical field and does not analyze the differences in thirst-quenching effects of commonly available beverages in the food sector. This study aims to fill this gap by comprehensively examining the differences in thirst-quenching effects among various beverages. We hypothesize that electrolyte drinks are more effective than other beverages in achieving thirst relief.

## 2. Experiment Section

### 2.1. Study Participants

This study recruited 13 male university students from Beijing (height: 173.15 ± 4.97 cm, weight: 63.20 ± 8.40 kg, BMI: 21.01 ± 1.94 kg/m^2^, body fat percentage: 16.33 ± 2.87%, age: 23.92 ± 1.14 years). The inclusion criteria were as follows: (1) body mass index (BMI) between 18.5 and 23.9 kg/m^2^; (2) regular aerobic exercise habits; (3) no clinically diagnosed diseases; (4) no smoking, excessive alcohol consumption, or other harmful lifestyle habits; (5) no participation in other clinical or nutritional research studies within one month prior to the experiment, with a commitment not to participate in any other exercise or nutrition intervention experiments during the study period; (6) willingness to voluntarily adhere to the experimental protocol. This study was approved by the Ethics Committee of the Institute for Food and Nutrition Development, Ministry of Agriculture and Rural Affairs (Approval No.: IFNDLLSC20240529), and all participants signed a written informed consent form.

### 2.2. Experimental Methods

#### 2.2.1. Preparations Before the Experiment

Three days prior to each exercise experiment, strict control is applied to the participants’ diet, fluid intake, and exercise activities. Participants are required to follow the dietary recommendations provided by this experiment, avoiding high-sodium foods, alcoholic beverages, and caffeinated drinks. They must also record their specific dietary intake during the experiment using a food diary. Additionally, participants’ daily water intake is maintained at approximately 1.7 L, and detailed records are kept. To ensure participants’ hydration status is within a normal range on the day of this experiment, they are instructed to avoid high-intensity exercise. Furthermore, participants must complete their last meal by 8:00 p.m. the evening before each experiment and consume 500 mL of water within 4 h before bedtime. Participants are required to fall asleep between 11:00 p.m. and 1:00 a.m.

On the day of the formal experiment, all participants wake up at 7:00 a.m. Morning urine is collected (to assess whether participants are in a normal hydration status for the day), followed by immediate consumption of 200 mL of drinking water. At 7:30 a.m., a standard breakfast is provided along with an additional 200 mL of drinking water.

#### 2.2.2. Experimental Design

This study employs a randomized crossover design. Each participant is required to complete five exercise sessions, each consisting of 30 min of rope skipping at a rate of 60 skips per minute. A 7-day washout period is applied between each experimental session. During these sessions, participants will randomly consume 6 mL per kg of body weight from one of five different beverages. The beverages used in this experiment were all commercially available drinks. Plain water, carbonated drink (containing 10.6 g of carbohydrates and 12 mg of sodium per 100 mL), fruit juice drink (containing 10.2 g of carbohydrates and 25 mg of sodium per 100 mL), electrolyte drink (containing 5.5 g of carbohydrates, 52 mg of sodium, 10–25 mg of potassium, ≥3 mg of calcium, ≥3 mg of magnesium, and 0.1 mg of vitamin B6 per 100 mL), and tea beverage (containing ≥360 mg of tea polyphenols per kg). The study design includes five groups corresponding to the type of beverage: the water supplementation group, the carbonated beverage supplementation group, the fruit juice supplementation group, the electrolyte beverage supplementation group, and the tea beverage supplementation group.

#### 2.2.3. Experimental Procedure

**Figure 1:**
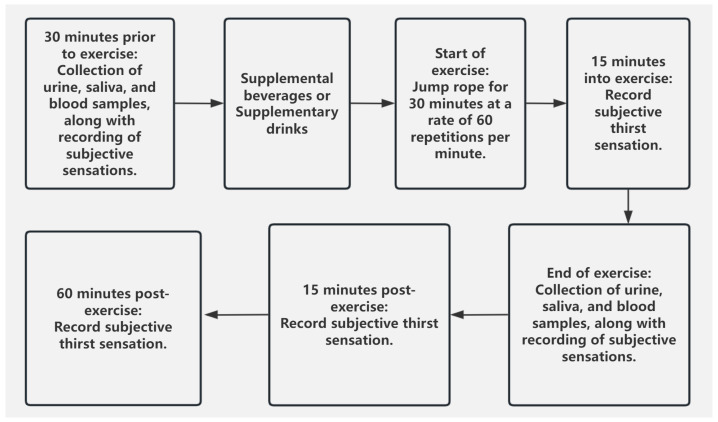
Experimental Flowchart.

On the experimental day, all participants woke up at 7:00 a.m., and a morning urine sample was collected to assess their hydration status for the day. Participants then immediately consumed 200 mL of drinking water. At 7:30 a.m., a standardized breakfast (prepared according to the “Dietary Guidelines for Chinese Residents 2022”, provided uniformly by the laboratory) and 200 mL of drinking water were served. One hour after consuming breakfast, participants arrived at the laboratory (with the experimental environment temperature controlled at 26 ± 1 °C, relative humidity maintained at 55 ± 5%), rested for 10 min, and then provided urine, saliva, and venous blood samples. Net body weight was measured (participants were asked to remove clothing and wipe off any excess moisture from their bodies), and subjective thirst levels were recorded. Afterward, participants were randomly assigned to consume one of the experimental beverages as required. Ten minutes later, they began performing a 30 min jump rope exercise at a rate of 60 jumps per minute. Subjective thirst levels were recorded at the start of the exercise, at 15 min, at the end of the exercise, 15 min post-exercise, and 60 min post-exercise. Physiological indicator samples were collected after the exercise, and net body weight was measured again. The above process is illustrated in Figure 1 for better clarity and understanding.

#### 2.2.4. Sample Information

Water was regular drinking water. The carbonated beverage was a slightly acidic drink containing dissolved carbon dioxide, with 10.6 g of carbohydrates and 12 mg of sodium per 100 mL. The juice beverage was apple juice, containing 10.2 g of carbohydrates and 25 mg of sodium per 100 mL. The electrolyte beverage was a slightly acidic drink containing 5.5 g of carbohydrates, 52 mg of sodium, 10–25 mg of potassium, ≥3 mg of calcium, ≥3 mg of magnesium, and 0.1 mg of vitamin B_6_ per 100 mL. The tea was a green tea beverage with polyphenol content ≥360 mg/kg.

#### 2.2.5. Testing Indicators and Methods

Venous blood parameters, including serum sodium, potassium, calcium, chloride ions, hematocrit (Hct), and hemoglobin (Hb), were assessed with a biochemical analyzer (Sorvall ST 40, Thermo Fisher, Waltham, MA, USA), an automated blood rheology analyzer (SA-6000, Scinco, Seoul, South Korea), and a hematology analyzer (XS-800I, Sysmex, Kobe, Japan). Urine and saliva osmolarity were determined using an automatic freezing point osmometer (FM-8P, Kyoto Electronics Manufacturing Co., Ltd., Kyoto, Japan). Body weight was measured with the Inbody 270 body composition analyzer (Inbody, Seoul, South Korea). Plasma volume changes were calculated using the hemoglobin (Hb) and hematocrit (Hct) values obtained through an established formula. [7].

(Hb_pre_/Hb_post_) × (1 − Hct_post_)/(1 − Hct_pre_) × 100% − 100% (1)

Thirst is a relatively subjective sensation, and due to individual differences in thirst perception thresholds, it is difficult to establish a standardized approach for objective evaluation. Currently, commonly used tools for thirst assessment in clinical settings include the Visual Analogue Scale (VAS), the Numerical Rating Scale (NRS), the Thirst Distress Scale (TDS), and the Perioperative Thirst Distress Scale (PTDS). Each of these tools has specific applications, focusing on different aspects of thirst evaluation depending on the clinical context [8]. In this study, a combination of existing methods is employed, incorporating a comprehensive assessment of sensations in the lips, throat, tongue, and oral cavity. Volunteers are asked to rate their thirst from 1 to 10, where a lower score indicates a higher level of thirst.

### 2.3. Statistical Analysis

Data were organized and analyzed using SPSS 27.0 software. The data are presented as mean ± standard deviation (Mean ± SD). First, the Shapiro–Wilk test was performed to assess the normality of the data, and the results indicated that all data followed a normal distribution. Therefore, a two-way repeated measures analysis of variance (ANOVA) was conducted to analyze the main effects of group and time, as well as their interaction. Bonferroni post-hoc tests were applied, and if an interaction effect was found, a simple effects analysis was further conducted.

## 3. Results

### 3.1. Effects of Pre-Exercise Supplementation with Different Beverages on Physiological Indicators

As shown in Table 1 and Table 2, body weight significantly decreased post-exercise in all five beverage supplementation groups (*p* < 0.05). The body weight post-exercise in the electrolyte drink group and the juice drink group was significantly lower than their pre-exercise body weight (*p* < 0.05). However, there were no significant differences between the five groups (*p* > 0.05).

Regarding serum Na^+^ concentrations, all groups showed a significant decrease post-exercise (*p* < 0.05). The tea drink group and the electrolyte drink group exhibited significant differences in serum Na^+^ concentrations post-exercise (*p* < 0.05). Regarding the change in serum Na^+^ concentrations from pre- to post-exercise, the electrolyte drink group showed significant differences compared to the other four groups (*p* < 0.05). Serum K^+^ concentrations increased post-exercise in all groups, but only the electrolyte drink group showed a significant increase in serum K^+^ concentrations post-exercise (*p* < 0.05). The changes in serum Ca^2+^ concentrations before and after exercise did not show a consistent trend; however, the serum Ca^2+^ concentrations in the electrolyte drink group and juice drink group were significantly higher post-exercise than pre-exercise (*p* < 0.05). Additionally, there were significant differences in serum Ca^2+^ concentrations post-exercise between the tea drink group and the electrolyte drink group, as well as between the tea drink group and the juice drink group (*p* < 0.05). Significant differences were also observed in the changes in serum Ca^2+^ concentrations from pre- to post-exercise between the tea drink group and the electrolyte drink group, as well as between the tea drink group and the juice drink group (*p* < 0.05). As for serum Cl^−^ concentrations, all groups except the electrolyte drink group showed a significant decrease post-exercise compared to pre-exercise (*p* < 0.05).

Regarding changes in plasma volume, all five fluid supplementation groups resulted in a decrease in plasma volume. Significant differences were observed between the electrolyte drink group and the carbonated drink group, the tea drink group, and the juice drink group (*p* < 0.05). Significant differences were also found between the water group and the tea drink group (*p* < 0.05).

Regarding urine osmolality, there was no consistent pattern in the changes in urine osmolality from pre- to post-exercise. The urine osmolality post-exercise in the water group and the electrolyte drink group was significantly lower than pre-exercise (*p* < 0.05), while the urine osmolality post-exercise in the carbonated drink group and the tea drink group was significantly higher than pre-exercise (*p* < 0.05). Significant differences in urine osmolality post-exercise were found between the water group and the carbonated drink group, the water group and the tea drink group, the water group and the electrolyte drink group, the water group and the juice drink group, the carbonated drink group and the electrolyte drink group, the tea drink group and the electrolyte drink group, and the electrolyte drink group and the juice drink group (*p* < 0.05). Additionally, significant differences were also observed in the changes in urine osmolality from pre- to post-exercise between the groups (*p* < 0.05).

Regarding saliva osmolality, saliva osmolality significantly increased post-exercise in all groups (*p* < 0.05). There was a significant difference in saliva osmolality post-exercise between the water group and the electrolyte drink group (*p* < 0.05). In terms of the changes in saliva osmolality from pre- to post-exercise, significant differences were observed between the tea drink group and the water group, as well as between the electrolyte drink group and the juice drink group (*p* < 0.05).

### 3.2. Effects of Pre-Exercise Supplementation with Different Beverages on Subjective Thirst Levels

As shown in Table 3, regarding subjective thirst levels, the scores in all groups initially decreased and then increased. At the pre-exercise (Pre-exercise), 15 min of exercise (15 min of exercise), and post-exercise (Post-exercise) time points, the differences in scores between the groups were not significant (*p* > 0.05). However, at 15 min post-exercise (15 min post-exercise), significant differences were found between the water group and the carbonated drink group, the water group and the juice drink group, the carbonated drink group and the tea drink group, as well as between the tea drink group and the juice drink group (*p* < 0.05). At 60 min post-exercise (60 min post-exercise), significant differences were also observed between the water group and the carbonated drink group, the water group and the juice drink group, the carbonated drink group and the tea drink group, and the carbonated drink group and the electrolyte drink group (*p* < 0.05).

## 4. Discussion

### 4.1. Main Finding of the Study and Comparison with Similar Previously Published Papers

#### 4.1.1. Weight

In the experiment, five groups of volunteers were supplemented with 6 mL/kg body weight of water, carbonated drink, juice drink, electrolyte drink, and tea drink before exercise, and they performed 30 min of rope skipping at a rate of 60 skips per minute in an environment with a temperature of 26 ± 1 °C and a relative humidity of 55 ± 5%. Changes in body weight can reflect hydration status, and post-exercise weight loss is widely considered an indicator of the degree of dehydration [3]. The experimental results showed that all five groups of volunteers experienced a decrease in body weight after exercise. The study by Cheuvront SN et al. [9] pointed out that a weight fluctuation of less than 1% indicates that the weight change is within the normal range, which aligns with the results of this experiment. However, no significant differences were observed between the groups, possibly due to the insufficient exercise duration, which may have prevented the effects of pre-exercise beverage supplementation on body weight from being fully reflected.

#### 4.1.2. Serum Electrolyte Concentration

Due to the crucial role of electrolytes in maintaining cellular function and hydration, their status can reflect the body’s hydration status. Therefore, changes in electrolyte levels can serve as an important physiological indicator for assessing hydration status [10]. During exercise, with significant fluid loss, particularly the loss of electrolytes in sweat, electrolyte imbalances may occur, triggering the body’s thirst response. In terms of changes in serum ion concentrations, this study found that both serum Na^+^ and Cl^−^ concentrations decreased to varying degrees. This may be because Na^+^ and Cl^−^ are the primary cation and anion in sweat, respectively [11]. During exercise, the body loses water and electrolytes through sweating, particularly sodium ions and chloride ions. Due to the significant dehydration caused by the exercise protocol used in this study, Na^+^ and Cl^−^ ions were lost through sweat, leading to a decrease in serum Na^+^ and Cl^−^ concentrations post-exercise. The electrolyte drink group showed the smallest fluctuation in serum Na^+^ concentration before and after exercise, with significant differences compared to the other four groups. This suggests that electrolyte drinks can provide the body with additional Na^+^, effectively reducing serum Na^+^ loss caused by exercise. Supplementing with an electrolyte drink before exercise can help alleviate fluctuations in serum Na^+^ concentration. Regarding serum Cl^−^ concentration, except for the electrolyte drink group, the serum Cl^−^ concentrations in the other four groups were significantly lower post-exercise compared to pre-exercise (*p* < 0.05). This indicates that electrolyte drinks can provide additional Cl^−^, effectively reducing serum Cl^−^ loss caused by exercise, and supplementing with an electrolyte drink before exercise can effectively mitigate the reduction in serum Cl^−^ concentration. Additionally, while serum K^+^ concentrations slightly increased post-exercise, serum Ca^2+^ concentrations showed no consistent trend in changes before and after exercise, and there were no clear patterns in the group differences. This suggests that the loss of K^+^ and Ca^2+^ during exercise is relatively small, but due to substantial water loss, there is an increase in serum ion concentrations. Valmir Oliveira Silvino et al. [3] suggested that electrolyte drinks have beneficial effects on electrolyte balance and physical performance, helping to moderately delay fatigue and maintain the body’s electrolyte balance. Electrolyte drinks containing electrolytes not only replenish fluids but also help sustain electrolyte balance. These findings are consistent with the results of this experiment.

#### 4.1.3. Plasma Volume

A deficiency in plasma volume can lead to decreased venous pressure, elevated body temperature, and dehydration [12]. Therefore, changes in plasma volume can be used as an indicator to assess the degree of dehydration and, in turn, the physiological state of thirst. In terms of plasma volume changes, all five rehydration strategies resulted in a decrease in plasma volume. However, the electrolyte drink group experienced the smallest reduction in plasma volume, with significant differences observed between this group and the carbonated drink group, tea drink group, and juice drink group. This suggests that electrolyte drinks are more effective in maintaining plasma volume stability. A significant difference was also found between the water group and the tea drink group, but no clear pattern was observed. Zhang Zhen et al. [13] selected three rehydration strategies: saline solution, electrolyte water, and water. They found that plasma volume decreased after exercise in all three groups. Compared to water supplementation, the decrease in plasma volume was significantly smaller in the electrolyte water group, which is consistent with the results of this experiment. Hiroshi Yamada et al. [14] found in their study with monkeys that fluctuations in plasma volume can affect body weight changes. In this experiment, both plasma volume and body weight decreased after exercise, which is consistent with the results of this study.

#### 4.1.4. Subjective Thirst Perception

Thirst sensation is regulated by the central nervous system, which perceives changes in hydration status. Dehydration is typically associated with changes in osmolarity and volume of the fluid system. Osmolarity changes are detected by the organum vasculosum of the lamina terminalis (OVLT), while volume changes are signaled through the hypothalamus to stimulate thirst and the release of vasopressin. Changes in blood volume are detected by atrial pressure receptors, further triggering drinking behavior [15]. This study found that the changes in thirst perception followed a trend of first decreasing and then increasing. At the start of this exercise, the thirst levels across all groups were similar. Thirst reached its highest point at the end of the exercise and 15 min after, and although it somewhat alleviated 60 min post-exercise, it had not yet returned to baseline levels. Studies have shown that there is a close relationship between thirst perception and fluid intake behavior, with fluid intake often being guided by the sensation of thirst. The primary purpose of fluid intake is to maintain internal balance by replenishing the fluids lost during exercise [16]. However, subjective thirst perception is not always equivalent to bodily dehydration that triggers drinking behavior. Thirst perception can sometimes be influenced by subconscious or subjective factors [17]. The water group performed the best in relieving subjective thirst, with significant differences observed between it and some other groups. This suggests that in the public’s subjective perception, water is more effective at quenching thirst despite a certain discrepancy when compared with objective physiological indicators. This result is consistent with the conclusions of Catherine Peyrot des Gachons and others [18].

### 4.2. Strengths and Limitations

This study comprehensively analyzed the differences in thirst-quenching effects of commonly available beverages on the market, providing a valuable reference for real-life exercise hydration practices. However, the small sample size in this study limits the generalizability of the results. Individual differences among various populations, such as gender, age, and exercise habits, may influence how different beverages affect thirst relief.

### 4.3. New Directions for the Future Research

Future research could expand the sample size to include individuals of different genders, ages, and exercise habits in order to improve the generalizability of the results. Since thirst perception and hydration needs may vary across different populations, increasing the sample size would help to more accurately assess the thirst-quenching effects of beverages.

## 5. Conclusions

This study compared and analyzed the differences in thirst-quenching effects of various beverages when consumed before exercise. The supplementation of electrolyte drinks showed better results in maintaining the balance of objective indicators during exercise. However, traditional thirst is a subjective experience, and sufficient fluid intake can reduce the sensation of thirst during exercise. Water is perceived as the most effective drink for quenching thirst when people subjectively feel thirsty, which does not align with our initial hypothesis. It is worth noting that the changes in subjective thirst perception after consuming different beverages do not always correlate directly with changes in physiological indicators. This suggests that in the process of fluid intake, both subjective perceptions and objective indicators should be evaluated comprehensively, which warrants further research in this area.

## Figures and Tables

**Table 1:** Effects of Pre-exercise Supplementation with Different Beverages on Physiological Indicators (Pre- and Post-exercise Values) (*n* = 13).

Variable	Group	PRE	POST	*F_Time_*	*F_Group_*	*F_Time_* × *_Group_*
Weight (kg)	W	63.28 ± 8.95	63.02 ± 8.87	173.756 *	0.001	0.458
	C	63.06 ± 8.25	62.85 ± 8.19			
	T	63.32 ± 8.65	63.05 ± 8.61			
	E	63.18 ± 8.13	62.92 ± 8.09 ^#^			
	J	63.19 ± 8.21	62.98 ± 8.18 ^#^			
	*F*	0.002	0.001			
	*p*	1	1			
Serum Na^+^ Concentration (mmol/L)	W	141.27 ± 4.34	134.99 ± 6.36 ^#^	295.454 *	0.460	14.211 *
	C	140.87 ± 2.72	133.25 ± 3.35 ^#^			
	T	139.96 ± 2.85	131.12 ± 3.33 ^#d^			
	E	138.02 ± 3.78	136.89 ± 3.90 ^#c^			
	J	141.02 ± 3.46	135.00 ± 5.93 ^#^			
	*F*	1.891	2.704			
	*p*	0.124	0.039			
Serum K^+^ Concentration (mmol/L)	W	5.01 ± 0.69	5.09 ± 0.66	11.827 *	0.586	1.814
	C	4.87 ± 0.73	5.03 ± 0.73			
	T	4.96 ± 0.38	4.97 ± 0.37			
	E	4.83 ± 0.42	5.16 ± 0.36 ^#^			
	J	4.72 ± 0.31	4.83 ± 0.29			
	*F*	0.599	0.665			
	*p*	0.817	0.519			
Serum Ca^2+^ Concentration (mmol/L)	W	2.27 ± 0.18	2.21 ± 0.34	3.587	2.424	4.680 *
	C	2.26 ± 0.17	2.38 ± 0.27			
	T	2.29 ± 0.13	2.16 ± 0.35 ^de^			
	E	2.32 ± 0.17	2.49 ± 0.19 ^#c^			
	J	2.35 ± 0.24	2.50 ± 0.26 ^#c^			
	*F*	0.521	3.851			
	*p*	0.721	0.008			
Serum Cl^−^ Concentration (mmol/L)	W	96.75 ± 3.56	96.04 ± 4.03 ^#^	40.179 *	0.214	0.736
	C	97.02 ± 5.23	96.046 ± 5.40 ^#^			
	T	97.68 ± 3.93	96.81 ± 3.55 ^#^			
	E	97.50 ± 3.39	97.14 ± 3.94			
	J	96.51 ± 3.89	95.74 ± 4.52 ^#^			
	*F*	0.191	0.247			
	*p*	0.942	0.911			
Urine Osmolarity (mOsm/kg)	W	1022.72 ± 124.75	853.45 ± 108.70 ^#bcde^	0.146	9.004 *	19.965 *
	C	1120.25 ± 114.30	1189.82 ± 129.81 ^#ad^			
	T	1086.98 ± 84.63	1162.65 ± 89.87 ^#ad^			
	E	1055.26 ± 96.65	996.78 ± 114.96 ^#abce^			
	J	1070.1 ± 120.97	1132.015 ± 139.18 ^ad^			
	*F*	1.431	18.517			
	*p*	0.235	<0.001			
Salivary Osmolality (mOsm/kg)	W	150.67 ± 45.21	170.53 ± 40.22 ^#^	205.213 *	1.900	6.420 *
	C	151.08 ± 28.03	180.43 ± 23.74 ^#^			
	T	160.38 ± 33.70	200.68 ± 22.92 ^#d^			
	E	145.61 ± 29.95	159.09 ± 27.42 ^#c^			
	J	165.05 ± 24.42	190.09 ± 30.21 ^#^			
	*F*	0.732	3.928			
	*p*	0.574	0.007			

“*” indicates *p* < 0.05; ^#^ *p* < 0.05 indicates a significant difference between PRE and POST; ^a^ *p* < 0.05, compared to the water supplementation group (W); ^b^ *p* < 0.05, compared to the carbonated beverage supplementation group (C); ^c^ *p* < 0.05, compared to the tea beverage supplementation group (T); ^d^ *p* < 0.05, compared to the electrolyte beverage supplementation group (E); ^e^ *p* < 0.05, compared to the fruit juice supplementation group (J).

**Table 2:** Effects of Different Beverages Supplemented Before Exercise on Changes in Physiological Thirst-Related Indicators Before and After Exercise (*n* = 13).

Variable	Group	Change During Exercise
Weight (kg)	W	−0.26 ± 0.19
	C	−0.21 ± 0.13
	T	−0.27 ± 0.15
	E	−0.26 ± 0.11
	J	−0.21 ± 0.15
	*F*	0.458
	*p*	0.766
Serum Na^+^ Concentration (mmol/L)	W	−6.28 ± 3.58 ^d^
	C	−7.62 ± 2.20 ^d^
	T	−8.83 ± 2.73 ^d^
	E	−1.14 ± 1.49 ^abce^
	J	−6.01 ± 3.46 ^d^
	*F*	14.211
	*p*	<0.001
Serum K^+^ Concentration (mmol/L)	W	0.08 ± 0.20
	C	0.16 ± 0.26
	T	0.01 ± 0.38
	E	0.33 ± 0.36
	J	0.11 ± 0.39
	*F*	1.814
	*p*	0.138
Serum Ca^2+^ Concentration (mmol/L)	W	−0.06 ± 0.31
	C	0.12 ± 0.20
	T	−0.13 ± 0.29 ^de^
	E	0.17 ± 0.14 ^c^
	J	0.15 ± 0.13 ^c^
	*F*	4.68
	*p*	0.002
Serum Cl^−^ Concentration (mmol/L)	W	−0.71 ± 0.66
	C	−0.92 ± 0.79
	T	−0.86 ± 1.25
	E	−0.36 ± 0.80
	J	−0.77 ± 0.99
	*F*	0.736
	*p*	0.571
Plasma Volume (%)	W	−0.05 ± 0.02 ^c^
	C	−0.07 ± 0.03 ^d^
	T	−0.09 ± 0.03 ^ad^
	E	−0.03 ± 0.03 ^bce^
	J	−0.07 ± 0.02 ^d^
	*F*	10.17
	*p*	<0.001
Urine Osmolarity (mOsm/kg)	W	−169.27 ± 95.72 ^bcde^
	C	69.57 ± 74.08 ^ad^
	T	75.67 ± 50.07 ^ad^
	E	−58.48 ± 39.14 ^abce^
	J	61.92 ± 138.08 ^ad^
	*F*	19.965
	*p*	<0.001
Salivary Osmolality (mOsm/kg)	W	19.86 ± 11.01 ^c^
	C	29.35 ± 14.56
	T	40.31 ± 21.55 ^ade^
	E	13.48 ± 6.28 ^c^
	J	25.05 ± 14.21 ^c^
	*F*	6.42
	*p*	<0.001

^a^ *p* < 0.05, compared to the water supplementation group (W); ^b^ *p* < 0.05, compared to the carbonated beverage supplementation group (C); ^c^ *p* < 0.05, compared to the tea beverage supplementation group (T); ^d^ *p* < 0.05, compared to the electrolyte beverage supplementation group (E); ^e^ *p* < 0.05, compared to the fruit juice supplementation group (J).

**Table 3:** Effects of Pre-Exercise Beverage Supplementation on Subjective Thirst Levels (*n* = 13).

Group	Pre- Exercise	15 Min of Exercise	Post- Exercise	15 Min Post- Exercise	60 Min Post-Exercise	*F* *_Time_*	*F_Group_*	*F_Time_* × *_Group_*
W	7.92 ± 1.59	5.92 ± 1.71	4.33 ± 1.49	4.92 ± 1.26 ^be^	6.08 ± 1.38 ^be^	119.709 *	12.187 *	1.341
C	7.85 ± 1.39	3.92 ± 2.50	2.92 ± 1.75	2.08 ± 1.66 ^ac^	3.23 ± 1.59 ^acd^			
T	7.94 ± 1.33	5.62 ± 1.45	4.23 ± 2.17	4.46 ± 1.33 ^be^	5.92 ± 1.93 ^b^			
E	7.52 ± 1.78	5.04 ± 2.11	4.15 ± 1.91	3.54 ± 1.39	5.31 ± 1.44 ^b^			
J	8.01 ± 1.52	4.54 ± 1.61	2.92 ± 1.61	2.59 ± 1.46 ^ac^	4.01 ± 2.47 ^a^			
*F*	0.201	2.315	2.097	0.271	6.152			
*p*	0.937	0.068	0.092	<0.001	<0.001			

“*” indicates *p* < 0.05; ^a^ *p* < 0.05, compared to the water supplementation group (W); ^b^ *p* < 0.05, compared to the carbonated beverage supplementation group (C); ^c^ *p* < 0.05, compared to the tea beverage supplementation group (T); ^d^ *p* < 0.05, compared to the electrolyte beverage supplementation group (E); ^e^ *p* < 0.05, compared to the fruit juice supplementation group (J).

## Data Availability

The original contributions presented in this study are included in the article. Further inquiries can be directed to the corresponding author.
